# Health Benefits of the Diverse Volatile Oils in Native Plants of Ancient Ironwood-Giant Cactus Forests of the Sonoran Desert: An Adaptation to Climate Change?

**DOI:** 10.3390/ijerph19063250

**Published:** 2022-03-10

**Authors:** Gary Paul Nabhan, Eric Daugherty, Tammi Hartung

**Affiliations:** 1 Southwest Center, University of Arizona, Tucson, AZ 85721, USA; eric13daugherty@gmail.com; 2 Desert Canyon Farm, Canon City, CO 81212, USA; desertcanyonfarm@gmail.com

**Keywords:** biogenic volatile organic compounds (BVOCs), deserts, climate change, essential oils, health benefits, Sonoran Desert

## Abstract

We document the species richness and volatile oil diversity in Sonoran Desert plants found in the Arizona Uplands subdivision of this binational USA/Mexico region. Using floristics, we determined that more than 60 species of 178 native plants in the ancient ironwood-giant cactus forests emit fragrant biogenic volatile organic compounds (BVOCs), especially with the onset of summer monsoons. From these desert species, more than 115 volatile oils have been identified from one biogeographic region. For the 5 BVOCs most commonly associated with “forest bathing” practices in Asian temperate forests, at least 15 Sonoran Desert plant species emit them in Arizona Uplands vegetation. We document the potential health benefits attributed to each of 13 BVOCs in isolation, but we also hypothesize that the entire “suite” of BVOCs emitted from a diversity of desert plants during the monsoons may function synergistically to generate additional health benefits. Regular exposure to these BVOC health benefits may become more important to prevent or mitigate diseases of oxidative stress and other climate maladies in a hotter, drier world.

## 1. Introduction

The fragrance emitted by plants, also known as biogenic volatile organic compounds (BVOCs) found in fragrant volatile oils, are emitted by many species in the Sonoran Desert and other arid landscapes to protect them from damaging solar radiation, transpiration, and herbivory, all of which are likely to be aggravated by climate change. In addition, they provide a characteristic fragrance of “desert rain” relished by many who may choose to use it in commercially available therapeutic aerosol sprays, tinctures, and showers or feature it in “forest bathing” walking tours in the Southwest USA [1].

Many of these BVOCs can be readily absorbed by the human body through inhalation and register within the brain in as little time as 22 s, taking less than 90 more seconds for them to be released into the bloodstream [2,3]. Within half an hour’s time, they may be found present in every cell of the body and reach all the body’s organs [4]. It takes two and a half hours or less for most of therapeutical aerosol inhalation of volatile oils to be metabolized in ways that may potentially affect human health in a more lasting manner [4,5,6]. 

To understand the potential therapeutic benefits of these desert plant-based fragrances, let us venture into the Sonoran Desert where many claim that with the onset of monsoon rains, a feeling of elation comes as “the desert smells like rain” [1,7]. There, because of detailed floristic and biogeographic surveys that we have participated in over four decades, we can offer the first survey of the diversity of BVOCs found in one vegetation type or landscape. 

More than a decade ago, it was suggested that the volatile essential oils emitted by desert vegetation—also called phytoncides in some studies [8]—posed more risks than additional benefits to human health [9]. Yet, these same biogenic volatile organic compounds are amply documented in many studies to enhance human physical and psychological health in the face of stressful societal conditions (see reviews [10,11]). 

There has been pioneering work on BVOCs in the arid grassland edges of the Sonoran Desert near Biosphere II in Arizona by Guenther et al. [9] and Jardine et al. [12]. More generally, Rinnan et al. [13] have hypothesized that future scientific studies should focus their future inquiries on BVOC emissions in “extreme environments” such as deserts, for they are precisely where plant volatile emissions should be particularly intense relative to those in more temperate environments, “as these compounds may have important roles in stress resistance and adaptation to extremes.”

As desert ecologist Stephen Buchmann [14] has eloquently reminded us, many desert herbs and shrubs may be conceived of as “miniature chemical factories”. This is evident in one of the Sonoran Desert’s most common plants, *Larrea tridentata*, known in English as the creosote-bush which, according to Jardine et al. [12], emits more than 35 distinct monoterpenoid, sesquiterpinoids and other BVOCs, many of which have strong fragrances. Curiously, some of these biologically active volatiles such as trans-caryophyllene, are derived from the endophytic fungus, *Phoma* sp., which sometimes grows as a pathogen in creosote-bush [15]. Due to the pungently sweet, musty, medicinal turpentine odors of creosote-bushes’ most abundant BVOCs–isoprene, vinyl and methyl ketones–some desert residents chronically sense that it offers the characteristic odor of deserts after rain, or of the seashore [16,17]. However, this demographically dominant plant is by no means the only major emitter of BVOCs that give Sonoran Desert habitats their renowned fragrances.

In fact, Felger et al. [18] hypothesized that there are many other fragrant plants hiding under the skirts of creosote-bush in the Sonoran Desert’s Arizona Uplands: “Creosotebush *(Larrea tridentata)* and bursage (*Ambrosia dumosa* and/or A. *deltoidea)* are among the most conspicuous perennials across most of the valley plains and expansive bajadas. Two words—mixed desertscrub—belie a potpourri of the hundreds of species on complex upland habitats”. It may be possible to use scientific literature accessible through Google Scholar to evaluate which desert plants may be among the many factors conferring potential health benefits to Sonoran Desert dwellers. 

## 2. Objectives

This study surveys the variety and potential utility of BVOCs found in the iconic “ancient ironwood-giant cactus forests” harbored in the Arizona Uplands subdivision of the Sonoran Desert of North America. While the ancient ironwood-giant cactus forests of the Sonoran Desert continue to be well-known for its powerfully uplifting fragrances emitted during its summer monsoon season [7,19], the diversity of fragrances from BVOCs in their floras have not yet been studied well enough to confirm that they have therapeutic value akin to those documented for Shinrin-Yoku practices [20]. While we assume that any health benefits accrued by walking in deserts after rains are multi-factorial and may be balanced by mediators such as other sensory or non-sensory stimulators, we wish to determine whether BVOC fragrances should or should not be further evaluated as one of the major contributing factors. 

Our objectives for this inquiry are to (1) provide an inventory of all the plants associated with the giant cactus-ancient ironwood forests in the Sonoran Desert for known BVOCs emitted from their oil-rich foliage, blossoms and bark; (2) determine which of the essential oils in two local Sonoran Desert floras emit the same secondary compounds as those documented in the forest floras of Shinrin-Yoku parks in Korea and Japan [10,21]; (3) identify any studies which confirm which, if any, particular BVOCs play roles in generating the healthful effects; (4) project BVOC diversity in a single arid landscape or vegetation; (5) speculate on their potential roles in adapting to climate change.

Antonelli et al. [10] have suggested that just five BVOCs found in species of gymnosperms (such as the hinoki cypress) dominate the secondary chemical compounds which generate most of the significant health benefits supposedly associated with the Shinrin-Yoku forest parks and trail systems. Therefore, we will pay particular attention to the presence of five BVOCs including α-Pinene, β-Pinene, β-Myrcene, Camphene and D-Limonene, along with 8 other BVOCs in Sonoran Desert plants. However, we will also document the presence of roughly a hundred other fragrant BVOCs in the ancient ironwood and giant cactus forests of the Sonoran Desert. For thirteen particular BVOCs, we have surveyed their potential health benefits documented in refereed journal articles found through Google Scholar searches.

## 3. Materials and Methods

As yet, there is no “comparative biogeography of BVOCs” per se; therefore, we have chosen to build toward that by focusing on the subregion of the Sonoran Desert known as the Arizona Uplands. In it, we focus on the local floras of two areas protected by the USA National Park Service, where the ancient ironwood-giant cactus forests are a prevalent landscape. One is in Saguaro National Park, in the Tucson Mountains near Tucson, Arizona; the other is in Organ Pipe Cactus National Monument, a UNESCO Biosphere Reserve that includes the Ajo Mountains near Ajo, Arizona. Both are within 500 to 1500 m elevation and receive an annual rainfall of less than 250 mm/year [18,22]. The forests are structured around legume tree-dominated nurse plant anchored by ironwoods *(Olneya)*, mesquites *(Prosopis)* or palo verdes *(Parkinsonia).* They serve as “dark gap” regeneration sites for a mosaic of short trees, shrubs, subshrubs, cacti, succulent, ephemeralized annuals, perennial vines, geophytes, and grasses that grow in the shade of 50- to 500-year-old trees [23,24], just as “light gap” openings in tropical and temperate cloud forests serve as regeneration suites in wetter vegetation. Species found under these legume trees that represent several different plant growth forms and families emit volatile essential oils from their bark, foliage, floral buds, blossoms, and nectar. Since these legume tree canopies create microenvironments buffered from temperature extremes and winds [23], we have detected floral fragrances lingering longer in theses cooler and less exposed micro-climates under these shade trees for hours [25,26].

We first contacted a dozen career desert ecologists, botanists, horticulturists, nurserymen, and pollination ecologists to get their subjective lists of the most fragrant plants in the Sonoran Desert region. From the 68 species they suggested, we eliminated those outside the Arizona Uplands subregion of the Sonoran Desert and compared the remaining species with an inventory we had previously conducted of all plants known to be directly associated with nurse plant guilds under ironwood trees (*Olneya tesota*) in these 2 ancient ironwood-giant cactus forests [24]. We also added congeners—plants in the same genera as those on the list—that also occurred in the Arizona Uplands. These occurrences in this habitat type were compiled from field plots of desert ecological surveys of ironwood habitats by our team, compiled in Nabhan and Carr [27]. We then determined which of these plant species were confirmed in the detailed local floras from Arizona: The Saguaro National Park/Tucson Mountains flora of Rondeau et al. [22], updated on the Arizona Native Plant Society website, and the flora of Organ Pipe Cactus National Monument/Ajo Mountains of Bowers [28], updated by Felger et al. [18]. 

Through Google Scholar, we then accessed and read over 100 scientific articles from the last half century that identified secondary compounds that are emitted as BVOCs from Sonoran Desert species. In the Arizona Uplands. We used key words such as BVOCs, volatile oils, terpenes, and all five specific BVOCs (below) repeatedly noted in other studies. We also used McGee’s [17] encyclopedic compilation to link particular BVOCs to the characteristic fragrances. We then calculated the percentages of species in each flora that had potent BVOCs, and which had the five BVOCs (most repeatedly associated with the healthful benefits in the forest bathing literature; α-pinene, β-pinene, β-myrcene, camphene and D-limonene. Finally, we compile the scientific documentation of “potential” health benefits attributed to 13 dominant BVOCs in the Sonoran Desert plant species commonly found in the nurse plant guilds of Arizona Uplands vegetation. The obvious limitations of this kind of search are 2-fold: (1) phytochemical assays of the plants in discussion were accomplished from a wide variety of methodologies for many purposes: medicinal screening; pollinator feeding studies; air contamination studies; etc.; (2) few of the studies (other than those of pollination ecologists) distinguished floral BVOCs from herbal or foliage BVOCs. 

## 4. Results

We summarize our results in Table 1 and a short compilation of data in the other tables. At least 178 dicot plant species in the 2 southern Arizona floras have been documented to be associated with ancient ironwood-giant cactus guilds where ironwood (*Olneya tesota*) serves as a major nurse plant. Sixty-eight species in this Sonoran Desert nurse plant guild have floral or herbal (leaves, stems, bark) fragrances potent enough to be detected, and described by desert botanists, emitting secondary compounds [1]. Using the 68 species that have aromatic blossoms, foliage, or bark as the total for the ancient ironwood-giant cactus guild, the proportion found in each local flower survey is roughly the same: 48% at Organ Pipe Cactus National Monument/Ajo Mountains, and 50% at Saguaro National Park West/Tucson Mountains. These percentages are preliminary and are likely to rise as other botanists and lay readers of this article offer new documentation of notably aromatic desert plants.

Table 2 is a summary of the number of species from these two floras that have documented secondary compounds which are potentially emitted as BVOCs. While the laboratory methodologies identifying these secondary compounds are so varied that there is no way to use them to rank which of these species have more potent fragrances, they demonstrate that the ancient ironwood-giant cactus guild in southern Arizona harbors a remarkably diverse set of fragrances with its flora. More than one hundred different BVOCs are released from the volatile oils exuded onto the surfaces of leaves, branchlets, stems, trunks, roots, sepals, and petals of Sonoran Desert plants, and from the fragrances in their nectar. 

Table 3 allows us to discern what BVOCs of importance in Asian forest bathing therapy sites are also accessible in the Arizona Uplands of the Sonoran Desert. While we are particularly interested in 13 of the BVOCs overall, we focus on the 5 most important BVOCs or phytoncides frequently found along hinoki cypress forest trails where Shinrin-Yoku walking therapies are commonly practiced; they are all are emitted by the Arizona Uplands vegetation as well. The volatiles in this table are ranked using Antonelli’s order of those most potent in the forest atmosphere, with Sonoran Desert plant species used as examples for each: limonene fragrances were emitted by 12 species in ancient ironwood-giant cactus forests; α-pinene by 10 species; myrcene by 7 species; b-pinene by 6 species; camphene by 4 species. 

As noted above, some studies [9,12] have suggested that the isoprene, vinyl and methyl ketones and other terpenic-aromas from creosote-bush are the dominant BVOCs at the edge of the Sonoran Desert during the summer monsoon season. However, our communication with other desert botanists suggest that a suite of memorable fragrances is emitted from at least 66 species of desert plants in the Arizona Uplands just before or during the summer rains. These may be likened to an “orchestra” of fragrances that are inhaled “in concert” to generate a sensory effect greater than the sum of its parts. We use McGee’s [17] encyclopedic field guide as well as personal experience to empirically compare BVOCs to characteristic fragrance below.

One of us (T.H.) has spent their career growing herbs, detecting BVOCs, identifying and describing their fragrances to nursery customers. From her experience, the twelve desert plants with D-limonene may emit fragrances that can be harshly terpenic, citrus-like, reminiscent of orange peels or tart berries, pine needles or peppery herbs. The ten species with α -pinene offer warm, fresh pine-like, resinous aromas with sweet earthy and woody notes. The half-dozen species with β-pinene also have a strong pine needle scent, but offer a drier, woodier note, and sometimes green hay-like notes as well, as they do in basil, dill, parsley, roses, and rosemary. The seven species with myrcene emit a lavender-like aroma, that can also have woody, resinous, musky, citrus, green peppery, clove-like, terpenic, and fruity, red grape aromas [17]. The four species with camphene emit fragrances reminiscent of a pine forest, for they are dominated by camphor-like aromas, but also include damp, pungent, spicy medicinal, herbal notes reminiscent of mints, citrus and freshly cut wood.

Keep in mind that most desert plants emit distinctive aromas that integrate and balance two or more of the characteristic fragrances of a particular volatile oil blend. The flowers of desert willow (*Chilopsis linearis*) will remind one of the fragrances of sweet violets, but the leaves have a much more medicinal aroma. Odora (*Porophyllum gracile*) smells quite strong and reminds one of the smells of rue and cilantro mixed together, some people enjoy this fragrance, while others find it repugnant or unpleasant. The linalools in wingpod purslane *(Portulaca umbracticola)* are floral-like lavender, but with a note of orange citrus. The desert wolfberry (*Lycium andersonii*) contains limonene, which smells similar to orange peels. Oreganillo (*Aloysia wrightii)* has limonene and carvone oils, and when the carvone is an R-form, it will smell similar spearmint chewing gum or tea, but if it is a S-form, it will smell similar to the caraway seeds baked in rye bread.

This suite of no less than sixty desert plant fragrances emitted with the onset of monsoonal thunderstorms are far richer and more varied than the singularly pungent “turpentine” or solvent-like scent of creosote-bush [17]. Even the highly-recognized fragrance of creosote bush and its endophytes—collectively known in the herbal trade as *chaparral tea*—may be the synergistic emissions of some 35 distinct blend of terpenes and other BVOCs, and not “one fragrance” [12,15]. In Table 4, we highlight the health benefits documented from the emissions of 13 BVOCs present in the species comprising the ironwood nurse plant guild Sonoran Desert of North America, five of which are important to forest-bathing in the hinoki-dominated cold temperate forests in Asia. 

## 5. Conclusions

We have identified the health benefits potentially afforded from thirteen of the more than one hundred BVOCs emitted from the foliage and flowers of desert plants. They are particularly evident and pungently expressed during the monsoonal rainy season of the iconic “Sonoran Desert summer” [1,7,63]. They emit a rich menage of aromatic fragrant oils that desert dwellers somehow come to associate with “the smell of rain” itself. 

While we can only document the potential health benefits attributed to each of these 13 volatile oils from particular desert plants in isolation, we hypothesize that the entire orchestra or “entourage” of BVOCs emitted from a diversity of desert plants during the monsoons may function synergistically to generate additional health benefits.

These oily compounds may have evolved to reduce transpiration and herbivory on the foliage of desert plants or to attract pollinators and other floral visitors. However, when shifts in the ozone content, humidity, barometric pressure, wind speed and cation exchange capacity [64] all dramatically occur with the arrival of a monsoonal stormfront in the desert, these oils are volatilized and saturate the air. They are held in microclimates especially found in and near the shaded nurse plant guilds of the ancient ironwood-giant cactus forests of Arizona and Sonora. 

Curiously, this flush of volatile oils in the air often begins immediately prior to the rains, well before the raindrops wash these oils from the leaf surfaces of the desert plants. Even as torrential rains dilute these oils on the leaves, they persist in the desert atmosphere for another hour or more after the rains have ceased. Amazingly, there is some evidence that not only humans, but to other vertebrates (mammals such as camels) and invertebrates (arthropods such as fruit flies and springtails) also respond positively to the rain-like scent of geosmin and musty plant fragrances that are emitted with the onset of rains [65,66]. Geosmin in cactus flowers may be a lure for pollinators [17].

In short, there are brief flushes of volatility of these essential oils that are emitted in synchrony with summer monsoons that may confer health benefits not only to humans, but to other animals as well. However, due to low humidity, infrequent rains, and other abiotic factors during the drier months of the year, humans and others may be less exposed to these BVOCs, or their potency may be diluted.

We have hypothesized elsewhere that walking through or actively restoring plant cover in Sonora Desert may have physiological and emotional benefits to participants due to multiple factors [1,67]. As noted earlier, BVOCs from desert plants may in some manner contribute to improving sleep patterns, stabilizing emotional hormones, enhancing digestion, heightening mental clarity, and reducing depression or anxiety. While we are far from being able to compare whether potential benefits linger longer or are more intense in the hinoki forests of the Far East than in the ancient ironwood-cactus forests of the Sonoran Desert, we have established a biogeographic and floristic means of comparing BVOC diversity in different landscapes. To date, other studies have been remarkably vague in identifying the diversity of plant species or their densities in Korean and Japanese forest settings. 

With hotter, drier climates and more uncertain rainfall in the future, desert dwellers may experience more intense monsoonal thunderstorms that punctuate long drought spells [68]. As Rinnan et al. [13] have predicted, “climate warming is likely to significantly increase [B]VOC emissions from extreme environments both by direct effects on [B]VOC production and volatility, and indirectly by altering the composition of the vegetation [including microbes in soil crusts.].” Their use of VOCs in this particular case applies to BVOCs in their study. The kinds of unprecedented heat waves and drought durations now being suffered in the Sonoran Desert (as elsewhere) will likely increase the quantity of volatile oils that desert plants exude or excrete onto their leaves, as means to prevent aggravated water loss [69,70]. If so, when rain does arrive and washes off the accumulated aromatic oils on the foliage of desert plants, there may be an even greater dose of BVOCs emitted and retained within desert microenvironments. Some of these most certainly include the potentially healthful BVOCs identified in a preliminary manner in this study. 

Inhabitants of arid lands may more urgently require the very health benefits that are generated from frequent engagement with or immersion in the Sonoran Desert’s aromatic plants [67,71]. Since more of the earth’s land surface will be facing hotter and often drier conditions, it will be critical to understand the ways in which arid-adapted plants with pungent BVOCs may dampen or mitigate the many stresses on the human metabolism. Unless we engage in hands-on ecological restoration which regenerates such aromatic plants in greater abundance [71], it is likely that such metabolic stresses will become more frequent and intense as climatic changes advance.

## Figures and Tables

**Table 1:** Synopsis of Sonoran Desert flora and its percentage of plants with BVOCs.

Region	Area in Acres	Total Taxa in Flora	Number of Species in Ancient Ironwood-Giant Cactus Guild in So. Arizona	Number of Species with Odors Described in Floras	Number of Species with BVOCs Documented from Flowers or Foliage	Number of Species with the 5 BVOCs Most Active in Forest Bathing Health Benefits: α-Pinene, β-Pinene, β-Myrcene, Camphene, & D-Limonene
Organ Pipe Cactus N.M.	15,360	657	114	54–55 (48%)	23 (20.2%)	13 (11.4%)
Saguaro National Park West/Tucson Mountains	19,600	630	94	47–48 (50%)	20 (21.3%)	11 (11.7%)
Total	ca 35,000	—	178	66	26	15

**Table 2:** Summary of the number of species from these two floras that have documented essential oils (and other secondary compounds), with an “X” marking which plants have known chemicals which are potentially emitted as BVOCs.

Family	Species Known from Under Ironwood in Ancient Desert Forests in Arizona Uplands	Common Names (English & Spanish)	Contains Known BVOCs	Contains One or More of the 5 BVOCs Most Associated with Health Benefits	References
Adoxaceae	*Sambucus canadensis*	Elderberry	X		[29,30]
Amaranthaceae	*Atriplex canescens*	Fourwing saltbush	X	X	[31]
Asteraceae	*Ambrosia ambrosioides*	Ambrosia-leaf Bur-ragweed, canyon ragweed, ambrosia bursage, *chicura*	X	X	[32]
	*Ambrosia confertiflora*	Slimleaf bursage, weak-leaf bur-ragweed, *estafiate, istafiate*	X	X	[32,33]
	*Ambrosia deltoidea*	Triangle-leaf bursage, triangle bur ragweed, triangle bursage, *chicurilla, ambrosia, estafiate, chamizo forrajero*	X	X	[32]
	*Ambrosia dumosa*	White burrowbush, burro-weed, *chicurilla, estafiate*	X	X	[32]
	*Baccharis salicifolia*	Douglas’ false willow, mule-fat, seepwillow, *batamote, jarilla, hierba del pasmo*	X		[34]
	*Brickellia coulteri*	Coulter brickellbush	X		[35]
	*Encelia farinosa*	Brittlebush, *incienso*	X	X	[36]
	*Parthenium incanum*	Mariola, *hierba ceniza, hierba del guayule*	X	X	[37]
	*Perityle emoryi*	Emory’s rockdaisy, desert rock daisy, Emory’s rocklily	X		[38]
Burseraceae	*Bursera microphylla*	Elephant tree, *torote blanco*	X	X	[39,40]
Cactaceae	*Carnegiea gigantea*	Saguaro, giant cactus, *sahuaro*	X		[41]
	*Opuntia phaecantha*	Tulip pricklypear, dark-spined prickly pear, *nopal*	X		[42]
	*Peniocereus greggii*	Night-blooming cereus, desert queen-of-the-night, *sarramatraca*	X		[25]
Convolvulaceae	*Cuscuta sp.*	Dodder	X	X	[43]
Fabaceae	*Prosopis glandulosa*	Honey mesquite, *mezquite*	X		[44,45,46]
Lamiaceae	*Condea emoryi*	Desert lavender	X	X	[47]
Nyctaginaceae	*Mirabilis multiflora*	Colorado four o’clock	X	X	[48]
Onagraceae	*Oenothera arizonica*	Arizona evening primrose	X		[49]
Portulacaceae	*Portulaca oleracea*	Common purslane, *verdolaga*	X		[49]
Solanaceae	*Datura discolor*	Sacred datura	X		[25]
	*Datura wrightii*	Sacred datura, sacred thorn-apple	X	X	[25]
	*Lycium andersonii*		X	X	[31]
Verbenaceae	*Aloysia wrightii*	Lemon verbena, *oreganillo*	X	X	[50]
Zygophyllaceae	*Larrea tridentata*	Creosote, *hediondilla*	X	X	[12]

**Table 3:** Forest BVOCs and their chemical characteristics listed on the basis of the magnitude of emissions (in descending order, after Antonelli et al. [10]).

Molecule	Chemical Family	Sonoran Desert Plant Species
isoprene	Isoprenoids	*Larrea tridentata*
cis-3-hexen-1-ol	Green leaf volatiles	*Mirabilis multiflora*
cis-3-hexenal	Green leaf volatiles	*Larrea tridentata*, *Cylindoopuntia acanthocarpa*
cis-3-hexenyl acetate	Green leaf volatiles	*Mirabilis multiflora*
d-limonene	Monoterpene hydrocarbons	*Aloysia wrightii*, *Ambrosia confertifolia*, *A. dumosa*, *Bursera microphylla*, *Capsicum annuum*, *Condea emoryi*, *Cuscuta tuberculata*, *Encelia farinosa, Larrea tridentata*, *Lycium andersonii*, *Mirabilis multiflora*, *Parthenium incanum*
α-pinene	Monoterpene hydrocarbons	*Ambrosia ambrosioides*, *A. confertifolia*, *A. dumosa*, *Atriplex canescens*, *Bursera microphylla*, *Condea emoryi*, *Encelia farinosa*, *Larrea tridentata*, *Lycium andersonii*, *Parthenium incanum*
(E)-β-ocimene	Monoterpene hydrocarbons	*Ambrosia confertifolia*, *Datura wrightii*, *Larrea tridentata*, *Mirabilis multiflora*, *Parthenium incanum*
1,8-cineole	Monoterpenoid ethers	*Ambrosia ambrosioides*, *A. confertifolia*, *Condea emoryi*, *Datura wrightii*, *Parthenium incanum*
camphor	Monoterpenoid ketones	*Larrea tridentata*
linalool	Monoterpenoid alcohol	*Condea emoryi*, *Datura wrightii*, *Oenothera arizonica*, *Opuntia phaeacantha*, *Parthenium incanum*, *Portulaca umbracticola*, *Sambucus canadensis*
p-cymene	Aromatic monoterpene hydrocarbons	*Larrea tridentata*
sabinene	Monoterpene hydrocarbons	*Ambrosia ambrosioides*, *A. deltodea*, *A. dumosa*, *Bursera microphylla*, *Encelia farinosa*, *Parthenium incanum*
β-caryophyllene	Sesquiterpene hydrocarbons	*Ambrosia ambrosioides*, *A. confertifolia*, *A. deltoidea*, *A. dumosa*, *Bursera microphylla*, *Larrea tridentata*, *Parthenium incanum*, *Perityle emoryi*, *Prosopis glandulosa*
β-myrcene	Monoterpene hydrocarbons	*Ambrosia dumosa*, *Atriplex canescens*, *Bursera microphylla*, *Datura wrightii*, *Lycium andersonii*, *Parthenium incanum*
β-pinene	Monoterpene hydrocarbons	*Ambrosia ambrosioides*, *A. deltoidea*, *A. dumosa*, *Bursera microphylla*, *Lycium andersonii*, *Parthenium incanum*
β-3-carene	Monoterpene hydrocarbons	*Larrea tridentata*
borneol	Monoterpenoid alcohol	*Larrea tridentata*
bornyl acetate	Monoteropene-derived ester	
camphene	Monoterpene hydrocarbons	*Ambrosia confertifolia*, *Atriplex canescens*, *Larrea tridentata*, *Lycium andersonii*
terpinen-4-ol	Monoterpenoid alcohol	
α-copaene	Sesquiterpene hydrocarbons	
α-humulene	Sesquiterpene hydrocarbons	*Ambrosia ambrosioides*, *A. confertifolia*, *A. deltoidea*, *A. dumosa*
α-phellandrene	Monoterpene hydrocarbons	*Baccharis salicifolia*, *Carnegiea gigantea*
α-terpinene	Monoterpene hydrocarbons	*Ambrosia confertifolia*, *A. deltoidea*, *A. dumosa*, *Parthenium incanum*
α-terpineol	Monoterpenoid alcohol	
α-terpinolene	Monoterpene hydrocarbons	*Parthenium incanum*
β-phellandrene	Monoterpene hydrocarbons	*Carnegiea gigantea*
β-terpinene	Monoterpene hydrocarbons	*Ambrosia confertifolia*, *A. deltoidea*, *A. dumosa*, *Parthenium incanum*
(Z)-β-ocimene	Monoterpene hydrocarbons	*Ambrosia confertifolia*, *Datura wrightii*, *Larrea tridentata*, *Mirabilis multiflora*, *Parthenium incanum*
bergamotene	Sesquiterpene hydrocarbons	*Prosopis glandulosa*
DMNT	Homoterpene hydrocarbons	*Mirabilis multiflora*
longifolene	Sesquiterpene hydrocarbons	
methyl jasmonate	Jasmonate ester	
methyl salicylate	Benzoate ester	*Datura wrightii*, *Larrea tridentata*, *Mirabilis multiflora*, *Peniocereus greggii*
TMTT	Homoterpene hydrocarbons	
α-thujene	Monoterpene hydrocarbons	*Condea emoryi*
β-farnesene	Sesquiterpene hydrocarbons	*Ambrosia ambrosioides*, *A. confertifolia*, *A. deltoidei*, *A. dumosa*, *Prosopis glandulosa*

**Table 4:** Known Health Benefits of Volatiles Recorded in Sonoran Desert Nurse Plant Guilds.

BVOCs and Their Sonoran Desert Plant Examples	Known Benefit or Effect	References
D-limonene (Anderson Wolfberry, Brittlebush, Chiltepin, Colorado Four O’clock, Desert Lavender, Dodder, Creosote-bush, Elephant Tree, Mariola, Oreganillo, Weakleaf Bur Ragweed)	Antioxidant, antiproliferative, antidepressant, anti-inflammatory, antinociceptive, anxiolytic, blood pressure lowering, heart rate decrease	[10,51]
α-pinene (Anderson Wolfberry, Brittlebush, Creosote-bush, Desert Lavender, Mariola, Triangle-leaf bursage, Weakleaf Bur Ragweed, White Burrow-bush)	Antioxidant, anti-inflammatory, anxiolytic, antiproliferative, analgesic, sedative, neuroprotective, antidepressant, sleep improvement	[10,52]
β-pinene (Anderson Wolfberry, Elephant Tree, Mariola, Triangle-leaf Bursage, White Burrow-bush)	Antioxidant, antiproliferative, antidepressant, anti-inflammatory, anxiolytic, neuroprotective, sleep improvement	[10,52]
Myrcene (Anderson Wolfberry, Elephant Tree, Four-winged Saltbush, Mariola, Sacred Datura)	Analgesic, antiproliferative, anti-inflammatory, sedative, gastroprotective, myorelaxant, antidiabetic, antibacterial, anticancer, anticonvulsant	[10,53]
Camphene (Creosote-bush Four-winged Saltbush, Weakleaf Bur Ragweed)	Antioxidant, antinociceptive, antihyperlipidemic, antiproliferative, anti-inflammatory, anti-cancer, anti-fungal, anti-gastric ulcers	[10,54]
Caryophyllene (Canyon Ragweed, Elephant Tree, Emory’s Rockdaisy, Mariola, Weakleaf Bur Ragweed, White Burrow-bush)	Antioxidant, anti-inflammatory, antihyperglycemic, full agonist of cannabinoid receptor type 2, anti-microbial, chemopreventive, nephroprotective, cardioprotective, neuroprotective	[55]
Linalool(s) (Arizona Evening Primrose, Chiltepin, Creosote-bush, Desert Prickly Pear Cactus, Mariola, Purslane, Sacred Datura)	Antioxidant, neuroprotective, anti-inflammatory, anxiolytic, antidepressant	[52]
Sabinene (Brittlebush, Elephant Tree, Triangle-leaf Bursage, White Burrow-bush)	Antioxidant, antibacterial, anti-inflammatory, angiostatic, antiangiogenic, cytoprotective, anticancer	[56]
Cineole (Canyon Ragweed, Desert Lavender, Mariola, Mexican (Desert) Oregano, Sacred Datura)	Antioxidant, anti-inflammatory, mucolytic and spasmolytic in the respiratory tract, antiseptic, antimicrobial, gastrointestinal protective, hepatoprotective, analgesic, anti-nociceptive	[57]
Farnesene (Desert Prickly Pear Cactus, Honey Mesquite, Triangle-leaf Bursage, Weakleaf Bur Ragweed)	Antioxidant, neuroprotective, anti-inflammatory	[58]
Humulene (Brittlebush, Weakleaf Bur Ragweed, Triangle-leaf Bursage, White Burrow-bush, White Bursage)	Antioxidant, antibacterial, antibiofilm, anti-inflammatory, antitumor, gastroprotective, cicatrizing, analgesic	[59,60]
Ocimene (Brittlebush, Mariola, Weakleaf Bur Ragweed, Sacred Datura)	Analgesic, anti-inflammatory	[61]
Methyl Salicylate (Colorado Four O’clock Night-blooming Cereus Cactus, Sacred Datura, Sweet Acacia/Huisache)	Analgesic, antiseptic, anti-inflammatory	[62]

## Data Availability

Data available from G.P.N. and Erin Riordan, Desert Laboratory at Tumamoc Hill, University of Arizona.
